# Evaluation of helping babies breathe and essential care for every baby training in southern nations nationalities and people’s region, Ethiopia: applying a Kirkpatrick training evaluation model

**DOI:** 10.1186/s13104-020-05394-7

**Published:** 2020-12-17

**Authors:** Lalisa Chewaka Gamtessa, Firew Tiruneh Tiyare, Kindie Mitiku Kebede

**Affiliations:** 1 Department of Nursing, College of Medicine and Health Sciences, Mizan-Tepi University, Mizan-Aman, Ethiopia; 2 Department of Midwifery, College of Medicine and Health Sciences, Mizan-Tepi University, Mizan-Aman, Ethiopia; 3 Department of Public Health, College of Medicine and Health Sciences, Mizan-Tepi University, Mizan-Aman, Ethiopia

**Keywords:** Helping babies breathe, Essential care for ever baby, Knowledge, Training, Satisfaction, Ethiopia

## Abstract

**Objective:**

The aim of this evaluation was to assess the effectiveness of helping baby breathe (HBB) and essential care for every baby (ECEB) training program that has been implemented in southern nations nationalities and people’s region (SNNPR), Ethiopia.

**Result:**

The mean trainees’ satisfaction score was 32.88 (SD ± 2.68). The majority (93.88%) of the trainees scored ≥ mean. All trainees expressed that all parts of the training were important but the updated parts of the training were most useful to them. The mean knowledge score of trainees for HBB training increased from 64.42 (SD ± 17.43) before the training to 80.71 (SD ± 14.36) after the training. The increment was statistically significant at *p* < 0.001. For ECEB training, the mean knowledge score of the trainees was increased from 59.10 (SD ± 13.18) before the training to 73.73 (SD ± 14.17) after the training. The improvement was statistically significant at *p* < 0.001*.*

## Introduction

Globally, neonatal mortality declined by 51% between 1990 and 2017. Though the reduction was significant, it was estimated that 27.8 million neonates will die between 2018 and 2030 [1]. In Ethiopia, neonatal mortality rate (NMR) was high and accounted for 30 per 1000 live births in 2019 [2].

Studies have shown that locally adaptable trainings like ECEB and HBB can reduce neonatal and still birth rate [3–5]. The ECEB and HBB training was developed by the American Academy of Pediatrics (AAP) and its partners. The training intends to equip health care providers, with an optimum basic resuscitation skill and essential care after birth [6, 7]. This training significantly improved the knowledge and skill of health care providers in resource limited settings [8–10].

Moreover, study findings in resource limited countries showed that the training has resulted in a high level of satisfaction among the trainees [9, 11, 12]. Despite a high level of satisfaction, trainees recommended for extension of the training duration, incorporation of real life demonstrations, videos, and advanced resuscitation skills [9, 12].

ECEB and HBB training has been provided in Ethiopia since 2018. Nevertheless, the effect of this training on the level of trainees’ satisfaction, knowledge, and skill has not been yet reviewed. Evaluating training is as important as providing training because it helps to know the degree to which training objectives were achieved. Therefore, this evaluation was aimed to assess the effect of HBB and ECEB training on the satisfaction and knowledge of trainees in SNNPR using a Kirkpatrick training evaluation model [13].

## Main text

### Methods

#### Study setting and area

The evaluation was conducted on the training given in two Hospitals in SNNPR selected by the program facilitators. These are Mizan-Tepi University Teaching Hospital and Sawula General Hospital which are located at 585 km and 514 km away from Addis Ababa, respectively.

#### Design

A formative evaluation was conducted by applying the Kirkpatrick training evaluation model. The model is known well world-wide and comprises four levels. The four levels are reaction (level 1), learning (level 2), behavior (level 3), and result (level 4) [13]. Further detail about the Kirkpatrick model is presented as Additional file 1.

#### Trainers and trainees

The Village Health Partnership provided training of trainers for six senior lecturers on HBB and ECEB training. The six trainers (3 pediatric Nursing specialists, 2 General Practitioners, and 1 Clinical Midwife) provided the training for five rounds starting from 2018–2019.

A total of 98 health care providers encompassing nurses, midwives, anesthetists, and health officers who came from Gamo, Goffa, and Bech-Sheko Zones of SNNPR were trained. These trainees were those who were involved in newborn care, and selected by their respective health facilities. The training was given for four days by dividing the trainees into five sub-groups with a ratio of one trainer to five trainees as per the protocol.

#### HBB and ECEB training

HBB and ECEB training is an evidence based program developed by AAP and its partners to train health care providers to equip them with knowledge and proper skill of neonatal resuscitation in resource limited countries [6, 7]. The HBB training consists of four main lessons: preparation for birth, routine care, the Golden minute, and ventilation with normal or slow heart rate. The ECEB training addresses continuing skin to skin care, initiation of breast feeding, provision of eye and cord care, provision of vitamin K, examination of the baby, measurement of temperature, weighing, and classifying the baby [7]. The training contains action plan, provider guide, facilitator guide, flipcharts, and materials for demonstration.

#### Evaluation of knowledge

Following necessary orientations, a pre-test with 18 standardized multiple choice questions was given to assess the knowledge of trainees about the HBB course contents. Immediately after the completion of HBB training, the trainees were re-evaluated using similar 18 multiple choice questions used in the HBB pre-test. Similarly, before starting the ECEB training, a pre-test with 25 standardized multiple choice questions was given to assess the knowledge of trainees about ECEB course contents. After completion of the ECEB training, the trainees were re-evaluated using similar 25 multiple choice questions used in ECEB pre-test.

#### Evaluation of satisfaction

The trainees’ satisfaction was evaluated using seven standardized questions with five likert scales arranged from strongly disagree to strongly agree along with two open ended questions. The trainees’ were asked their satisfaction after completing the HBB and ECEB training.

#### Operational definitions

Knowledge: The immediate knowledge acquired as a result of the training measured by comparing the mean score of pre- and post-test results.

Satisfaction: Trainees who scored mean and above the mean were considered as satisfied but otherwise they were considered as unsatisfied.

### Data collection

Data were obtained from the reporting documents of HBB and ECEB training deposited at Mizan-Tepi University Educational Development Center (EDC). EDC is responsible for coordinating the HBB and ECEB training. The trainees’ profile, their knowledge and satisfaction scores are available at the EDC. Two trained BSc nurses collected data using the data extraction formats presented as Additional files 2, 3, 4, 5.

### Statistical analysis

Data were entered into Epi Info 7 and imported to SPSS version 21.0 for analysis. Descriptive statistics (frequencies and percentages) were computed to describe trainees’ socio-demographic characteristics and level of satisfaction. A Paired Samples T-test was used for comparing the pre-test and post-test mean knowledge score difference of the trainees. The statistical significance was determined at p-value < 0.05.

## Results

### Socio-demographic characteristics of trainees

More than two thirds of the trainees were females. The majority of trainees were nurses and midwives while anesthetists accounted for the smallest proportion of the trainees (Table 1).

**Table 1:** Socio-demographic characteristics of trainees in SNNPR, Ethiopia, 2018–2019

Variables		Frequencies	Percentages
Sex	Male	32	32.7
	Female	66	67.3
Health facility	Hospital	47	48.0
	Health center	51	52.0
Qualification	Degree	60	61.2
	Diploma	38	38.8
Professionals	Nurses	30	30.6
	Midwives	55	56.1
	Health officers	9	9.2
	Anesthetists	4	4.1

### Trainees’ satisfaction

The mean score of likert items used in evaluating satisfaction of trainees was 32.88. The majority of trainees scored ≥ the mean. All trainees mentioned that all parts of the training were important but they replied that the updated parts of the HBB and ECEB training were most useful for them. The majority of trainees were satisfied with the training, but a few trainees remained undecided on item 4 and item 7 (Table 2).

**Table 2:** Trainees satisfaction across items in SNNPR, Ethiopia, 2018–2019

	Items	Strongly agree	Agree	Undecided	Disagree	Strongly disagree
1	For the work, I do, the training was appropriate	74 (75.51%)	24 (24.49%)	0 (0.00%)	0 (0.00%)	0 (0.00%)
2	Training facilities and arrangements were satisfactory	67 (68.37%)	31 (31.63%)	0 (0.00%)	0 (0.00%)	0 (0.00%)
3	The facilitators were knowledgeable and skilled	67 (68.37%)	31 (31.63%)	0 (0.00%)	0 (0.00%)	0 (0.00%)
4	The facilitators were fair and friendly	60 (61.22%)	36 (36.74%)	2 (2.04%)	0 (0.00%)	0 (0.00%)
5	The Training updated my knowledge and skills	59 (60.20%)	39 (39.80%)	0 (0.00%)	0 (0.00%)	0 (0.00%)
6	Training objectives were met	73 (74.49%)	725 (25.51%)	0 (0.00%)	0 (0.00%)	0 (0.00%)
7	Teaching aids were useful	83 (84.69%)	12 (12.24%)	3 (3.06%)	0 (0.00%)	0 (0.00%)

### Trainees’ knowledge

The knowledge of the trainees improved after the training. For the HBB training, the mean knowledge score of the trainees was increased from 64.42 (SD ± 17.43) before the training to 80.71 (SD ± 14.36) after the training. This increment was statistically significant; t (97) = 11.146, *p* < 0.001. For the ECEB training, the mean knowledge score of the trainees was increased from 59.10 (SD ± 13.18) before the training to 73.73 (SD ± 14.17) after the training. This improvement was statistically significant; (t (97) = 11.684, *p* < 0.001.

## Discussion

Evaluation is very important to determine the effectiveness of programs. In the current evaluation, the HBB and ECEB training significantly improved trainees’ knowledge and nearly all trainees were satisfied with the training.

In this evaluation, trainees’ level of satisfaction was high. This finding is in line with several evaluation studies in resource limited countries [9, 12, 14]. Though majority of the trainees were satisfied with the training, a few trainees remained undecided on item 4 (facilitators were fair and friendly) and item 7 (teaching aids were useful). Therefore, to further enhance the trainees’ satisfaction, programmers and trainers should consider these two items. Moreover, all the trainees mentioned that the updated parts of HBB and ECEB training were most important. The updated parts include rational clinical practices related to suction, ventilation, cord care, and benefits of skin to skin [6, 7]. Therefore, focusing on these areas may further increase trainees’ satisfaction level.

The trainees’ knowledge score was also significantly improved immediately after the training. Several studies revealed that HBB and ECEB training significantly improved trainees’ knowledge [9, 10, 12, 14, 15].

## Conclusion

The HBB and ECEB training resulted in a significant increase in knowledge. Nearly all the trainees were satisfied by the training. More emphases should be given to the updated parts of the HBB and ECEB training as trainees expressed these parts were most useful.

## Limitations

The current evaluation exclusively focused on the level 1 (satisfaction) and level 2 (knowledge) of Kirkpatrick training evaluation model. We did not evaluate long-term effect of the training due to resource constraints. Furthermore, the findings of this study should be cautiously interpreted as the sample size was small.

## Supplementary Information

**Additional file 1**: The four levels of Kirkpatrick training Evaluation Model.

**Additional file 2**: Satisfaction tool.

**Additional file 3**: Data extraction checklists format.

**Additional file 4**: Knowledge check (HBB 2nd Edition).

**Additional file 5**: Essential Care for Every Baby (ECEB) Knowledge check.

## Data Availability

All the datasets used and or analyzed during the current study are available from a primary author on a reasonable request at lalisachewaka@gmail.com.

## Abbreviations

A.A: Addis Ababa
AAP: American Academy of Pediatrics
CI: Confidence interval
ECEB: Essential care for every baby
HBB: Helping babies breath
SD: Standard deviation
SNNPRs: South nations nationalities and peoples’ regions
