# Supplementary material for: Evaluation of helping babies breathe and essential care for every baby training in southern nations nationalities and people’s region, Ethiopia: applying a Kirkpatrick training evaluation model
Source: BMC Res Notes. 2020 Dec 17;13:567. doi: 10.1186/s13104-020-05394-7 (PMC7745724; doi:10.1186/s13104-020-05394-7)
Supplement: Supplementary file 1 — Additional file 1: The four levels of Kirkpatrick training Evaluation Model. [file 13104_2020_5394_MOESM1_ESM.docx]

**Additional file 2 – Satisfaction tool**

| **Items in smile sheet** | **Strongly Agree** | **Agree** | **Undecided** | **Disagree** | **Strongly Disagree** |
| --- | --- | --- | --- | --- | --- |
| 1. For the work, I do, the training was appropriate. |  |  |  |  |  |
| 2. Training facilities and arrangements were satisfactory. |  |  |  |  |  |
| 3. The facilitators were knowledgeable and skilled. |  |  |  |  |  |
| 4. The facilitators were fair and friendly. |  |  |  |  |  |
| 5. The Training updated my knowledge and skills |  |  |  |  |  |
| 6. Training objectives were met. |  |  |  |  |  |
| 7. Teaching aids were useful. |  |  |  |  |  |

1. What was the most useful part of the training course for you?

1. What part of the training course was useful to you?
