# Supplementary material for: Evaluation of helping babies breathe and essential care for every baby training in southern nations nationalities and people’s region, Ethiopia: applying a Kirkpatrick training evaluation model
Source: BMC Res Notes. 2020 Dec 17;13:567. doi: 10.1186/s13104-020-05394-7 (PMC7745724; doi:10.1186/s13104-020-05394-7)
Supplement: Supplementary file 2 — Additional file 2: Satisfaction tool. [file 13104_2020_5394_MOESM2_ESM.docx]

Additionals file 3: Data extraction checklists format

| No | Extracted items | | Frequency |
| --- | --- | --- | --- |
| 1 | Sex | Male |  |
|  |  | Female |  |
| 2. | Health facility | Hospital |  |
|  |  | Health centers |  |
|  |  | Others |  |
| 3. | Profession | Nurses |  |
|  |  | Midwives |  |
|  |  | Health officers |  |
|  |  | Anesthetist |  |
|  |  | Others |  |
| 4 | Qualification | Degree |  |
|  |  | Diploma |  |
|  |  | Others |  |
| 5 | Test score | Pre-test |  |
|  |  | Post-test |  |
