# Supplementary material for: Evaluation of helping babies breathe and essential care for every baby training in southern nations nationalities and people’s region, Ethiopia: applying a Kirkpatrick training evaluation model
Source: BMC Res Notes. 2020 Dec 17;13:567. doi: 10.1186/s13104-020-05394-7 (PMC7745724; doi:10.1186/s13104-020-05394-7)
Supplement: Supplementary file 3 — Additional file 3: Data extraction checklists format. [file 13104_2020_5394_MOESM3_ESM.pdf]

Essential Care for Every Baby (ECEB)
Knowledge check

Select the best answer to each question or statement.
Circle the letter of the correct answer.

1. How long should initial skin-to-skin care be provided by healthy mothers of well babies?
   a. At least one hour
   b. As long as it is convenient for the mother
   c. Only until the baby's temperature is normal
   d. At least 24 hours

2. During the first hour after birth, how often should babies be observed for breathing problems?
   a. Once during the hour
   b. Every 30 minutes
   c. Every 15 minutes
   d. Every 5 minutes

3. Why is it important to begin breastfeeding within the first hour after birth?
   a. It helps babies breastfeed more successfully
   b. It keeps the baby from crying
   c. It keeps babies alert soon after birth
   d. It helps babies breathe more easily

4. When should a baby be given liquids other than breast milk?
   a. When the baby does not feed at the breast within the first hour after birth
   b. When the mother has engorged breasts
   c. When the baby cries between feedings
   d. Never

5. What is an early sign that a baby is ready to breast feed?
   a. Crying for a long period of time
   b. Opening the mouth and licking
   c. Eyes closed and hands open
   d. Breathing rapidly with the mouth open

6. How fast should a normal baby breathe?
   a. At least 65 times per minute
   b. At any rate as long as there is no chest in-drawing
   c. 40-60 times per minute
   d. 25 times per minute

7. What is the most important reason for weighing all babies soon after birth?
   a. Birth weight may identify babies who need special care
   b. Mother and family members often want to know the birth weight
   c. Birth weight will determine how long a baby will breast feed at each feeding
   d. Birth weight will identify babies who need vitamin K

8. In what part of the eye should medicine to prevent eye infections be given?
   a. Inside the upper eyelid
   b. Inside the lower eyelid
   c. In the corner of the eye only
   d. On the outside of the eyelid

9. What is the normal temperature range for a healthy baby?
   a. 36.5°C - 37.5°C
   b. 34.0°C – 35.5°C
   c. 35.5°C - 36.5°C
   d. 37.5°C - 38.5°C

10. Which babies should be given vitamin K after birth?
    a. Only babies with bleeding
    b. Only babies with birth weight >2500 grams
    c. All babies
    d. Only sick babies

11. How should a baby be kept warm after skin-to-skin care?
    a. Bathing in warm water
    b. Wrapping in a clean, dry blanket or cloth
    c. Placing near warm stones
    d. Exposing to sunshine

12. How soon after birth can a healthy baby be bathed?
    a. As soon as the baby has a normal temperature
    b. As soon as normal breathing has been established
    c. At least six hours following birth
    d. Immediately after the first breastfeeding

13. What is a sign of good attachment at the breast?
    a. The baby's nose is pressed against the breast.
    b. The baby bites down and pulls on the nipple.
    c. Only the tip of breast is in the baby's mouth.
    d. The baby's mouth is wide open on the breast.

14. What should mothers do if they have breast engorgement?
    a. Feed more often or express milk
    b. Stop breastfeeding for one day
    c. Take antibiotics
    d. Apply herbs to the breasts

15. At 90 minutes after birth, a 2700 gram baby is skin-to-skin with the mother and has a temperature of 36.0 °C. What should you do next?
    a. Give antibiotics and seek advanced care.
    b. This temperature is normal and no special care is needed.
    c. Make the room warm and free of drafts and replace any wet clothing.
    d. Place warm stones next to the baby.

16. Babies with what problem might benefit from cup feeding?
    a. Vomiting with every feeding
    b. Unable to awaken for feedings
    c. Able to swallow but unable to suck effectively
    d. Unable to swallow

17. When cup feeding a baby, what is the correct action?
    a. Allow the baby to lick the milk from the cup
    b. Pour small quantities of milk into the baby's mouth
    c. Place the baby flat on his back
    d. Drip a continuous stream of milk into the mouth

18. Which of the following would describe convulsions?
    a. Occur only in the legs
    b. Cannot be stopped by holding arms and legs
    c. Occur in response to a loud noise
    d. Occur only when the baby is awake

19. When should a baby be treated with antibiotics?
    a. If birth weight is less than 2000 grams
    b. When a Danger Sign is present
    c. If the baby cries often
    d. If the baby appears to be in pain

20. When should the first dose of an antibiotic be given?
    a. After transfer for advanced care
    b. As soon as possible after a Danger Sign has been identified
    c. After all family members have been contacted
    d. At a time that is convenient for the health care provider

21. After the first day following birth, jaundice is severe when it appears on what body area?
    a. Back and abdomen
    b. White part of the eye
    c. Legs and arms
    d. Palms and soles

22. How often should a mother express milk for a baby who cannot feed at the breast?
    a. As often as the baby would normally feed (8-12 times per 24 hours)
    b. 3 times during the daytime only
    c. Once in the morning and once before bedtime
    d. 4 times per 24 hours

23. A 3000 gram baby is unable to suck or swallow during the first 6 hours after birth. How would you classify this baby and what should you do?
    a. The baby is normal, and breastfeeding should be encouraged
    b. The baby has a Danger Sign, and you should seek advanced care
    c. The baby has a feeding problem, and should be cup fed.
    d. The baby will probably die, and no further care should be provided

24. Which of the following is a sign that a baby is breastfeeding adequately?
    a. Crying within one hour after each feeding
    b. Vigorous sucking that causes nipple pain with each feeding
    c. Remains awake and active after each feeding
    d. Feeding 8 to10 times per day

25. You have determined that a baby with a birthweight of 1800 grams needs antibiotics. How much ampicillin (200 mg/mL) should you give?
    a. 0.6 mL
    b. 0.5 mL
    c. 1.0 mL
    d. 0.35 mL

Weight	**Ampicillin IM** Dose: 50 mg per kg every 12 hours<br>Add 2.5 ml sterile water to 500 mg vial - 200 mg/ml	**Gentamicin IM** **Dose:** 5 mg per kg every 24 hours if term; 4 mg per kg every 24 hours if preterm<br>20 mg per 2 ml vial - 10 mg/ml
**1.0 - 1.4 kg**	0.35 ml	0.5 ml
**1.5 - 1.9 kg**	0.5 ml	0.7 ml
**2.0 - 2.4 kg**	0.6 ml	0.9 ml
**2.5 - 2.9 kg**	0.75 ml	1.35 ml
**3.0 - 3.4 kg**	0.85 ml	1.6 ml
**3.5 - 3.9 kg**	1 ml	1.86 ml
**4.0 - 4.4 kg**	1.1 ml	2.1 ml
