# Supplementary material for: Evaluation of helping babies breathe and essential care for every baby training in southern nations nationalities and people’s region, Ethiopia: applying a Kirkpatrick training evaluation model
Source: BMC Res Notes. 2020 Dec 17;13:567. doi: 10.1186/s13104-020-05394-7 (PMC7745724; doi:10.1186/s13104-020-05394-7)
Supplement: Supplementary file 4 — Additional file 4: Knowledge check (HBB 2nd Edition). [file 13104_2020_5394_MOESM4_ESM.pdf]

Knowledge check (HBB 2nd Edition)

Select the best answer to each question or statement
Circle the letter of the correct answer

1. What should you do in The Golden Minute?
   a. Bathe the baby
   b. Deliver the placenta
   c. Evaluate the heart rate
   d. Help a baby breathe if necessary

2. To prepare for a birth
   a. You identify a helper and review the emergency plan
   b. You ask everyone but the mother to leave the area
   c. You prepare equipment only when you need it
   d. You do not need a helper

3 To prepare the area for delivery
   a. Open all the doors and windows to get fresh air
   b. Darken the room
   c. Make sure the area is clean, warm, and well-lighted
   d. Keep the room temperature cold

4. What should you do to keep the baby warm?
   a. Open all the windows
   b. Give the baby a bath after birth
   c. Place hot water bottles next to the baby's skin
   d. Place the baby skin-to-skin with mother

5. What should you do to keep the baby clean?
   a. Wash your hands before touching the baby and help mother wash her hands before breastfeeding
   b. Reuse the suction device before cleaning
   c. Keep the umbilical cord tightly covered
   d. Do not touch the baby

6. Which baby can receive routine care after birth?
   a. A baby who is not breathing
   b. A baby who is gasping
   c. A baby who is crying and/or breathing well
   d. A baby who is limp

7. Routine care for a healthy baby at birth includes
   a. Drying, removing the wet cloth, and bathing the baby
   b. Drying, removing the wet cloth, and positioning the baby skin-to-skin
   c. Bathing and putting clean clothes on the baby
   d. Drying and wrapping the baby in the wet cloth

8. When should the umbilical cord be clamped or tied and cut during routine care?
   a. After the placenta is delivered
   b. Around 1-3 minutes after birth
   c. Immediately after the baby is born
   d. Before a baby has cried

9. A baby is quiet, limp and not breathing at birth. What should you do?
   a. Dry the baby thoroughly
   b. Shake the baby
   c. Throw cold water on the face
   d. Hold the baby upside down

10. A newborn baby is quiet, limp and not crying. The baby does not respond to steps to stimulate breathing. What should you do next?
    a. Slap the baby's back
    b. Hold the baby upside down
    c. Squeeze the baby's ribs
    d. Begin ventilation

11. In which situation should a baby be suctioned?
    a. When a baby is crying at birth
    b. When a baby is crying but there is meconium in the amniotic fluid
    c. When you see secretions blocking the mouth and nose
    d. Before drying the baby

12. Suctioning a baby unnecessarily or frequently can
    a. Cause a baby to stop breathing
    b. Make a baby start coughing and breathing
    c. Stimulate a baby to cry
    d. Increase the baby's heart rate

13. Which of the following statements about ventilation with bag and mask is TRUE?
    a. The mask should cover the eyes
    b. Air should escape between the mask and face
    c. Squeeze the bag to produce gentle movement of the chest
    d. Squeeze the bag to give 80 to 100 breaths per minute

14. A baby's chest is not moving with bag and mask ventilation. What should you do?
    a. Stop ventilation
    b. Reapply the mask to get a better seal
    c. Slap the baby's back
    d. Give medicine to the baby

15. You can stop ventilation if
    a. A baby is blue and limp
    b. A baby's heart rate is slow
    c. A baby's heart rate is normal and the chest is not moving
    d. A baby's heart rate is normal and the baby is breathing or crying

16. A newborn baby's heart rate should be:
    a. Faster than your heart rate
    b. Slower than your heart rate
    c. Checked before drying the baby
    d. Checked only when the baby is crying

17. A baby who received ventilation
    a. Needs continued observation with mother
    b. Cannot be fed
    c. Always needs advanced care
    d. Should immediately receive antibiotics

18. When should the bag and mask and suction device be disinfected?
    a. After every use
    b. Only when they appear dirty
    c. Weekly
    d. Once a month
