# Supplementary material for: Evaluation of helping babies breathe and essential care for every baby training in southern nations nationalities and people’s region, Ethiopia: applying a Kirkpatrick training evaluation model
Source: BMC Res Notes. 2020 Dec 17;13:567. doi: 10.1186/s13104-020-05394-7 (PMC7745724; doi:10.1186/s13104-020-05394-7)
Supplement: Supplementary file 5 — Additional file 5: Essential Care for Every Baby (ECEB) Knowledge check. [file 13104_2020_5394_MOESM5_ESM.docx]

**Additional file 1: The four levels of Kirkpatrick training Evaluation Model**

**Level 1(Reaction)**: Measures how participants react to the training. An example of level 1measuremnt is satisfaction measured by items in the smile sheet.

**Level 2(Learning**): Measures whether trainees truly understood the training or not. Examples of level 2 measurements are knowledge skills measured by providing pre- and post-test.

**Level 3 (Behavior)**: This level measures whether the trainees utilize knowledge and skill of the program taught at the workplace. The level analyzes the differences in the participant’s behavior at work after completing the program usually 3–6 months after training.

**Level 4 (Results)**: This level determines if the material had a positive impact on the organization. Commonly regarded as the primary goal of the program, level four determines the overall success of the training by measuring factors different factors. A decrease in neonatal mortality due to change in skill at clinical practice is an example of level four.
